# Image-Guided Percutaneous Ablation for Primary and Metastatic Tumors

**DOI:** 10.3390/diagnostics12061300

**Published:** 2022-05-24

**Authors:** Arian Mansur, Tushar Garg, Apurva Shrigiriwar, Vahid Etezadi, Christos Georgiades, Peiman Habibollahi, Timothy C. Huber, Juan C. Camacho, Sherif G. Nour, Alan Alper Sag, John David Prologo, Nariman Nezami

**Affiliations:** 1 Harvard Medical School, Boston, MA 02115, USA; arianmansur@hms.harvard.edu; 2 Division of Vascular and Interventional Radiology, Russell H Morgan Department of Radiology and Radiological Science, The Johns Hopkins Hospital, Baltimore, MD 21287, USA; tgarg3@jhmi.edu (T.G.); cgeorgi@jhmi.edu (C.G.); 3 Division of Gastroenterology and Hepatology, The Johns Hopkins Hospital, Baltimore, MD 21287, USA; ashrigi1@jhmi.edu; 4 Division of Vascular and Interventional Radiology, Department of Diagnostic Radiology and Nuclear Medicine, University of Maryland School of Medicine, Baltimore, MD 21201, USA; vahid.etezadi@umm.edu; 5 Department of Interventional Radiology, The University of Texas MD Anderson Cancer Center, Houston, TX 77030, USA; phabibollahi@mdanderson.org; 6 Vascular and Interventional Radiology, Dotter Department of Interventional Radiology, Oregon Health and Science University, Portland, OR 97239, USA; huberti@ohsu.org; 7 Department of Clinical Sciences, College of Medicine, Florida State University, Tallahassee, FL 32306, USA; juan.camacho@radpartners.com; 8 Vascular and Interventional Radiology, Radiology Associates of Florida, Sarasota, FL 34239, USA; 9 Department of Radiology and Medical Imaging, Florida State University College of Medicine, Gainesville, FL 32610, USA; sherif.g.nour@gmail.com; 10 Division of Vascular and Interventional Radiology, Department of Radiology, Duke University Medical Center, Durham, NC 27710, USA; alan.sag@duke.edu; 11 Division of Vascular and Interventional Radiology, Department of Radiology and Imaging Sciences, Emory University School of Medicine, Atlanta, GA 30322, USA; john.david.prologo@emory.edu; 12 Experimental Therapeutics Program, University of Maryland Marlene and Stewart Greenebaum Comprehensive Cancer Center, Baltimore, MD 21201, USA

**Keywords:** ablation, image-guidance, primary, metastatic, tumors

## Abstract

Image-guided percutaneous ablation methods have been further developed during the recent two decades and have transformed the minimally invasive and precision features of treatment options targeting primary and metastatic tumors. They work by percutaneously introducing applicators to precisely destroy a tumor and offer much lower risks than conventional methods. There are usually shorter recovery periods, less bleeding, and more preservation of organ parenchyma, expanding the treatment options of patients with cancer who may not be eligible for resection. Image-guided ablation techniques are currently utilized for the treatment of primary and metastatic tumors in various organs including the liver, pancreas, kidneys, thyroid and parathyroid, prostate, lung, bone, and soft tissue. This article provides a brief review of the various imaging modalities and available ablation techniques and discusses their applications and associated complications in various organs.

## 1. Introduction

Image-guided percutaneous ablation is defined as the process of percutaneously introducing needlelike applicators to destroy or shrink tumors in a controlled and targeted fashion under image guidance. This approach is a well-established minimally invasive practice for primary and metastatic tumors [1,2]. The advent of image-guided percutaneous ablation along with transarterial therapies has allowed interventional radiologists to not only be involved in the diagnosis of cancer but also serve as key players in its treatment and follow-up care [3]. Imaging modalities to guide ablation probes range from ultrasound (US) to magnetic resonance imaging (MRI), and ablation modalities can be divided into several types depending on the form of energy delivery and tissue injury [4]. Given the imaging of cancerous tissues and the damage to the tissue architecture with ablation, this work is applicable to other fields, both within and outside of medicine, that employ deformational analysis [5,6,7]. This article reviews the current role of image-guided percutaneous ablation modalities in the treatment of malignancies in different organs.

## 2. Imaging Modalities for Guiding Percutaneous Ablation Devices

Imaging plays critical roles in the pretreatment diagnosis and monitoring of tumors, peri-treatment placement of the ablation probe, guiding chemical or energy deposition during the procedure, and post-treatment assessment of outcomes [8]. Therefore, the selection of a proper imaging modality is critical for successful ablation. US and computerized tomography (CT) are most commonly employed [9].

### 2.1. Ultrasound

US is one of the most readily available imaging modalities used for guiding percutaneous ablation devices. It is relatively easy to operate, does not impart ionizing radiation, and provides real-time, multiplanar guidance at a low cost [10,11]. However, the use of US is limited by significant operator dependency, difficulty targeting deep structures in patients with obesity, limited visualization of air containing organs (e.g., intestinal loops), and limited utility for the visualization of tumors such as hepatic or iso-echoic renal tumors [9,10,11,12,13]. Contrast-enhanced ultrasound (CEUS), in which microbubble contrast agents are used as acoustic enhancers, is an alternative modality that enhances procedure guidance and pre- and post-procedural evaluation [14,15]. While CEUS can enhance the visibility of small tumors at a relatively low-cost, it cannot detect all lesions (e.g., deeply seated lesions in the hepatic dome), requires specific operator experience, is limited by the number of contrast injections during each session, and is not widely available [10,16].

### 2.2. Fluoroscopy

Fluoroscopy is an imaging technique that employs the use of X-ray pulses to capture real-time moving images [17]. Fluoroscopy was the main imaging technique for percutaneous biopsies and drainage procedures before the advent of CT that slowly replaced its use [18]. While useful for providing real-time feedback, fluoroscopy is restricted by its limited ability to navigate out of its plane and the exposure to radiation to both the patient and operator [17].

### 2.3. Computed Tomography, Cone-Beam CT, and CT Fluoroscopy

CT is the most commonly used imaging modality to guide percutaneous ablation devices as it is widely accessible, has a wide field of view, and is not limited by bowel gas [19]. It is commonly used without contrast; however a limited dose of contrast may be administered to visualize the lesion or identify critical vital structures to avoid non-target injury. The primary disadvantages of CT are ionizing radiation, single plane acquisition (though newer technologies such as IMACTIS CT-Navigation™ System (Hinckley, UK) by BVM Medical, Hinckley, UK are simulating and providing multiplanar views), and limited real-time visualization of iso-dense targets, and requires caution with the use of contrast media in patients with renal insufficiency [9].

Cone-beam computerized tomography (CBCT) is another modality that is well-suited to provide high spatial resolution and 3D image reconstructions. However, CBCT is limited by the relatively longer acquisition time than conventional CT, which can introduce motion artifacts [17]. Fluoroscopy guidance can be paired with CT to provide real-time feedback. It can allow for faster procedural times and improve targeting accuracy by avoiding errors due to patients’ movement and breathing [17]. However, this technique provides a relatively high amount of radiation exposure to both the patient and operator [17]. Other ways to guide percutaneous ablation devices is through the use of lipiodol or radiopaque beads. These are sometimes used in transarterial chemoembolization and can enhance intraprocedural imaging guidance by their ability to be visualized with fluoroscopy or CT [20,21]. Other ways to guide percutaneous ablation devices is via pre-ablation embolization of lesions using lipiodol or radiopaque beads. These are used in transarterial chemoembolization and can enhance intraprocedural imaging guidance by their ability to be visualized by fluoroscopy or CT [20,21].

### 2.4. MRI

Magnetic resonance imaging (MRI) is less frequently used than US or CT but is useful in that it uses no ionizing radiation, provides a high contrast resolution between soft tissues, displays small tumors with increased sensitivity, allows for imaging in any orientation and on any plane, monitors thermal effects, and can be combined with diffusion-weighted imaging or MRI contrast agents to visualize more difficult lesions [11]. The primary advantage of MRI guidance for tumor ablation, however, lies in its unique thermal sensitivity that allows online monitoring of the progress of ablation. The main disadvantages of MRI use include the lack of MR compatible tools, closed MRI not having real-time guidance, a relatively complicated operation, susceptibility to artifacts, and high cost [11].

## 3. Physics and Mechanism of Action of Percutaneous Ablation Devices

**Figure 1:**
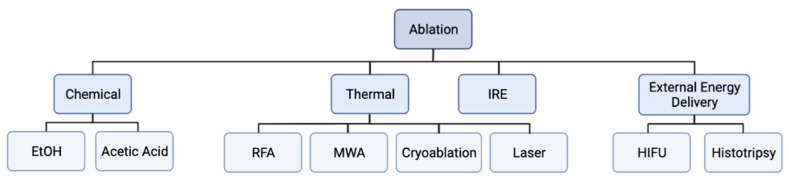
**Flowchart of ablation modalities.** Ablation modalities can be subdivided into thermal and non-thermal based modalities. Thermal ablation modalities include radiofrequency ablation (RFA), microwave ablation (MWA), cryoablation, and laser ablation. Non-thermal ablation modalities include chemical ablation (ethanol and acetic acid ablation), irreversible electroporation (IRE), and external energy delivery modalities (high intensity focused ultrasound (HIFU) and histotripsy).

The tumor ablation techniques are divided into chemical ablation, thermal ablation, irreversible electroporation, or external-energy-delivery-based ablation (Figure 1). Chemical ablation includes nonenergy-based ablation techniques such as intratumoral ethanol and acetic acid injection that cause coagulation necrosis leading to tumor ablation [1,22,23]. Thermal ablation includes modalities that destroy tissue via heat or cold and include radiofrequency ablation (RFA), cryoablation, microwave ablation (MWA), and laser ablation. External-energy-delivery-based ablation includes high-intensity frequency ultrasound (HIFU) and histotripsy (Figure 1). The advantages and disadvantages of the common ablation techniques are summarized in Table 1.

### 3.1. Radiofrequeny Ablation

**Figure 2:**
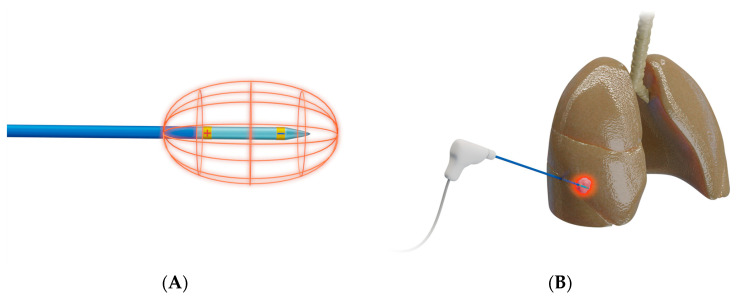
**Schematic presentation of radiofrequency ablation**. (**A**) Design of a bipolar RFA system, in which electrical current is delivered through an electrode that functions as both anode and cathode [26]; bi-polar electrodes obviate the need for grounding pads; (**B**) Representative example of RFA used to target a lesion in right middle lobe of a lung.

RFA (Figure 2) works by delivering radiofrequency waves in the 375–500 kHz range to an area surrounding a generator-coupled electrode, causing an oscillating electric field that creates frictional energy by electron collision. This collision generates heat that leads to eventual tumor destruction by coagulation necrosis from temperatures above 60 °C [19,24,25].

### 3.2. Microwave Ablation

**Figure 3:**
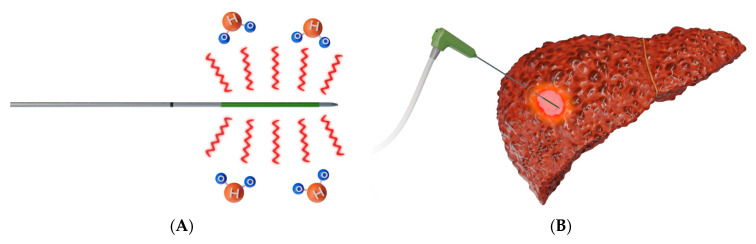
**Schematic presentation of microwave ablation.** (**A**) Design of a microwave antenna that generates an oscillating electromagnetic field, leading to the continuous realignment of molecules with an intrinsic dipole moment (mostly water) [26]; (**B**) Representative example of MWA used to target a liver tumor.

MWA (Figure 3) uses microwave energy in the 300–3000 MHz range from an antenna, creating oscillation of ions in the target [19]. This oscillation creates heat and results in coagulative necrosis. This technique allows for faster ablation times, larger ablation zones, and a reduced heat sink effect compared to RFA [27,28].

### 3.3. Cryoablation

**Figure 4:**
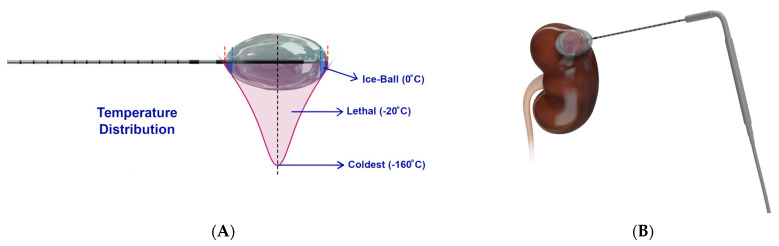
**Schematic presentation of cryoablation**. (**A**) Design of a cryoprobe, whereby a cryogen circulates to rapidly cool the cryoprobe to −160 °C, resulting in an ice ball around the cryoprobe of varying temperatures, of which −20 °C is lethal [26]. Figure adapted from Nelson et al. [26]; (**B**) Representative example of cryoablation used to target a kidney tumor.

Cryoablation (Figure 4) destroys tumors by applying freezing temperatures or alternating freeze–thaw cycles [29]. This technique works by utilizing the Joule–Thomson effect by which certain gases such as nitrogen, nitrous oxide, or argon drop in temperature when going from high pressure to low pressure [24,30]. Once the target region reaches temperature of −40 °C, ice crystals form in the extracellular space that leads to increased tonicity and osmotic damage to cells in the area [24,31,32]. Eventually, intracellular ice forms, rupturing the plasma and organelle membranes, followed by indirect cell death by thrombosis of damaged blood vessels that lead to ischemia and inflammation [24,33].

### 3.4. Irreversible Electroporation

**Figure 5:**
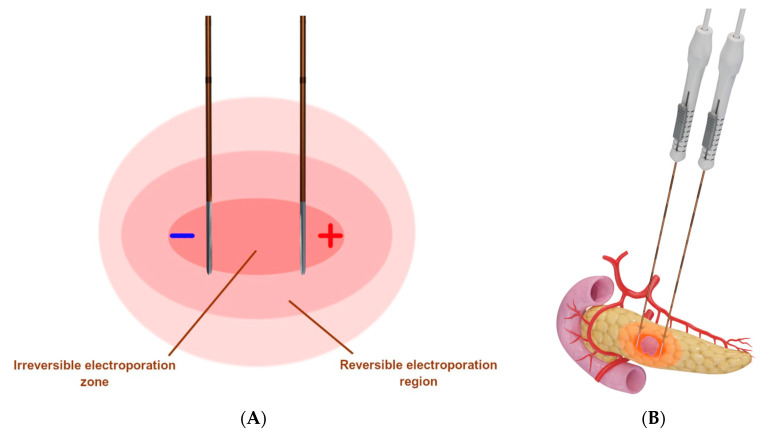
**Schematic presentation of irreversible electroporation**. (**A**) Two IRE probes are placed in parallel around the tumor, whereby multiple high-voltage electrical pulses are delivered to produce a uniform zone of ablation [26]; (**B**) Representative example of IRE used to target a pancreatic tumor.

IRE (Figure 5) is a nonthermal ablation modality that causes cell death through repeated short-duration high-voltage electrical pulses, leading to ruptured cellular membranes and irreversible cell death [34,35]. IRE differs from the thermal modalities in that it is not affected by the heat sink effect [1].

### 3.5. Laser Ablation

Laser ablation uses light energy to precisely heat a tumor electromagnetically and cause coagulative necrosis. It is a flexible technique that can be conveniently coupled through optical fibers that are MR compatible, but the small ablation zones require multiple placements [19]. Furthermore, laser ablation can have limited energy penetration as most body tissues absorb and scatter light [36].

### 3.6. High-Intensity Frequency Ultrasound

**Figure 6:**
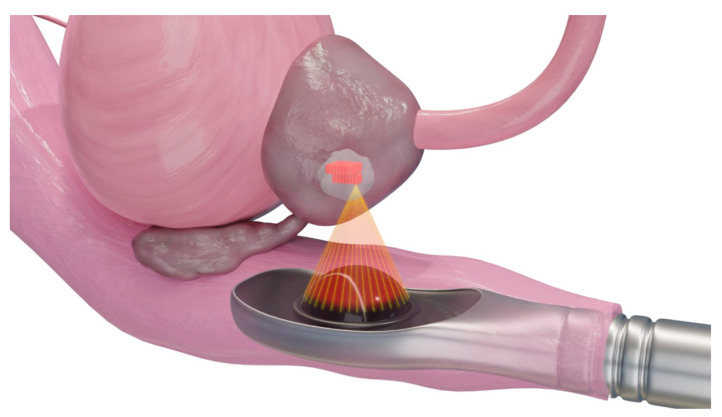
**Schematic presentation of high-intensity frequency ultrasound for ablation of a prostate tumor**. The extracorporeal transducer generates high-intensity ultrasound to carry and propagate energy onto a focused area deep within tissue media. Temperatures are raised to a level that is sufficient for thermotherapeutic effects, resulting in coagulation necrosis [38,39].

HIFU (Figure 6) is a noninvasive thermal ablation technique that causes coagulation necrosis by delivering high-intensity ultrasound waves onto a focal area [37]. The sources of ultrasound are often placed extracorporeally or rectally without the need for the transcutaneous insertion of probes.

### 3.7. Histotripsy

**Figure 7:**
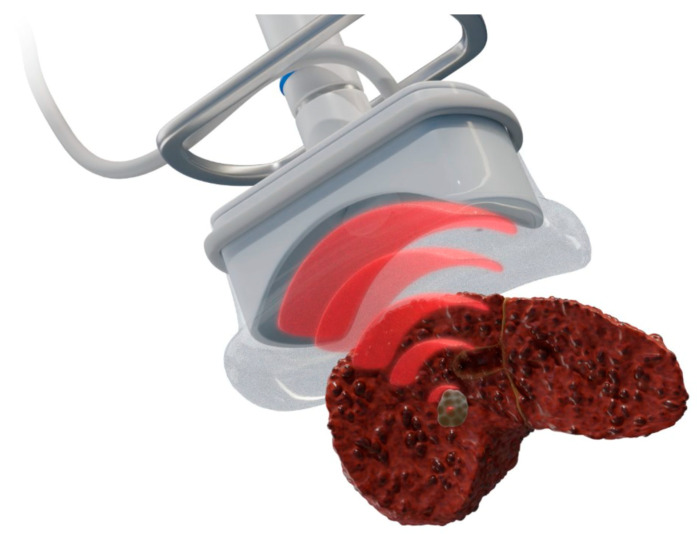
**Schematic presentation of histotripsy for liver tumor ablation**. Like HIFU, a focused transducer is used to generate and focus ultrasound to the tumor, but it instead causes mechanical damage without thermal coagulation via short, intense acoustic pulses that create dense energetic “bubble clouds” in the tissue media. These bubbles rapidly expand and collapse to mechanically disintegrate tissue [42].

Histotripsy (Figure 7) is a noninvasive ablation technique based on ultrasound but is nonthermal. It uses focused ultrasound waves to mechanically destroy tissue through cavitation, causing minimal damage to surrounding tissue [40]. It works by using pulsed sound waves to create “bubble clouds” from gases naturally occurring in the target. These bubbles form and collapse quickly, leading to mechanical force-based destruction of the tissue [41].

## 4. Primary and Metastatic Liver Tumors

**Figure 8:**
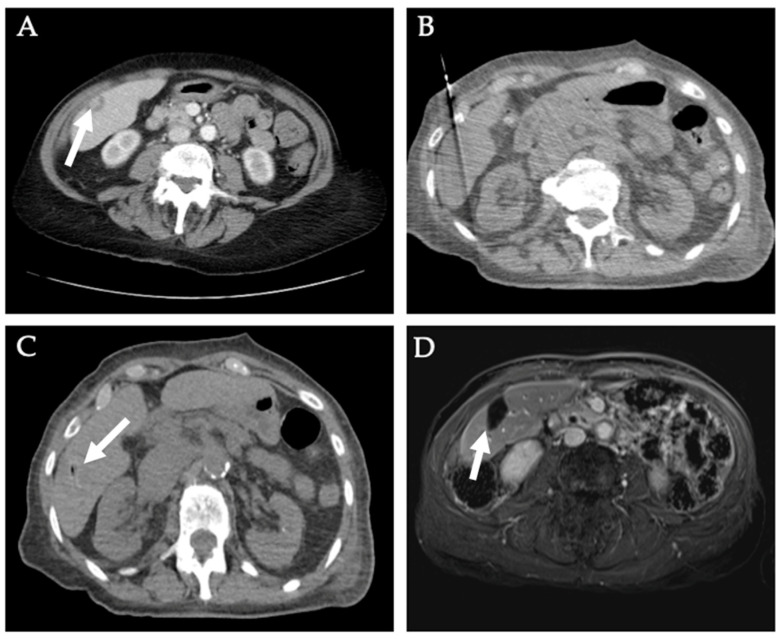
**Microwave ablation of hepatic metastatic leiomyosarcoma**. A 73-year-old female patient with a 2.1 × 1.5 cm sub-capsular metastatic leiomyosarcoma (white arrow) in segment V (**A**) on CT scan. The patient underwent chemotherapy followed by MWA. (**B**) Probe placement and (**C**) immediate post-ablation image of the right hepatic lobe lesion. The patient then underwent proton therapy of inferior vena cava mass and left hepatic metastasis. (**D**) Six-month follow-up MRI shows complete response.

The preferred treatment option for localized hepatocellular carcinoma (HCC) is surgical resection. However, a proportion of patients are not candidates for surgery due to the extent of the tumor or underlying poor liver function [43]. Ablation for localized HCC is a viable treatment option in patients with nonresectable tumors who are not transplant candidates, especially in patients with no worse than Child-Turcotte-Pugh class A or B cirrhosis, or in transplant candidates as a form of bridging or downstaging therapy [44]. The 2022 Barcelona Clinic Liver Cancer guidelines now recommend ablation as the standard of treatment in patients with solitary HCC ≤ 2 cm without vascular invasion or extrahepatic spread, for which liver transplant is not feasible [45]. Multiple percutaneous ablation modalities are used in treating liver tumors with the main techniques being RFA and MWA. MWA has several theoretical advantages over RFA in that it can obtain shorter ablation times, multiple ablations can be performed simultaneously, it can reach higher intratumoral temperatures with larger ablation zones, its efficacy is not reduced by electrical impedance, and it is less susceptible to the heat sink effect [44,46,47,48,49,50,51]. However, these advantages may make MWA more dangerous in injuring nearby structures [44,51,52]. Several randomized trials have been conducted to compare MWA versus RFA for HCC treatment, showing that MWA is comparable to RFA for HCC treatment in terms of safety and efficacy but has reduced local tumor progression and shorter ablation times (Table 2) [13,51,53,54,55,56,57,58,59]. A case of a patient receiving MWA for a hepatic metastatic leiomyosarcoma in segment V is presented in Figure 8.

Cryoablation can also be used in the treatment of HCC. While heat-based thermal ablation modalities are typically used in cirrhotic patients due to their lower rates of bleeding complications, cryoablation technology now includes thinner probes and helium–argon as a cryogen instead of liquid nitrogen [60,61,62,63]. Cryoablation is usually the preferred modality for hepatic tumors near vulnerable structures as it can produce smaller ablation zones, which can be monitored in real-time via intraprocedural CT [63,64]. Furthermore, cryoablation allows for neurolysis, making it suitable for subcapsular lesions or lesions close to the diaphragm. While the data comparing cryoablation to RFA for HCC is not as extensive as that between MWA and RFA, a randomized controlled trial and large population-based retrospective study found non-inferior results for cryoablation [63,65,66].

**Figure 9:**
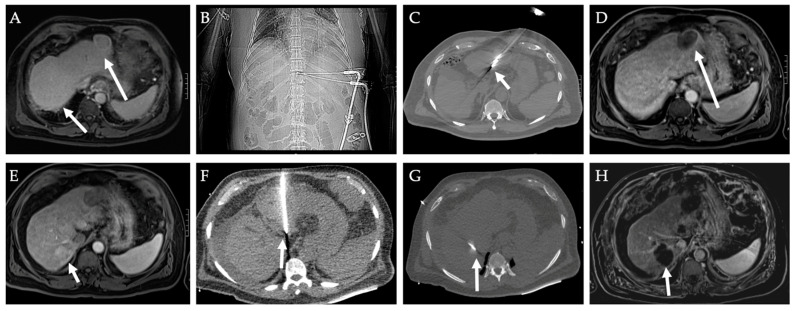
**Microwave ablation and irreversible electroporation of hepatocellular carcinoma**. A 72-year-old male with alcohol-induced cirrhosis and HCC was found to have a 3.5 × 2.6 cm biopsy-proven HCC lesion (white arrow) in segment II (**A**), LI-RADS 4 lesion in segment VII, and LI-RADS 3 lesion in the caudate lobe on MRI. (**B**,**C**) The patient underwent percutaneous MWA of the segment II HCC. (**D**) Four-month follow-up MRI showed complete response of the segment II HCC to MWA, but the two previously noted lesions (**E**) in the caudate lobe and segment VII progressed to LI-RADS 5. The patient underwent image-guided percutaneous ablation of these two lesions two months later, receiving (**F**) NanoKnife IRE for segment II/caudate and (**G**) MWA for segment VII lesion. (**H**) One-month follow-up MRI shows no residual tumor. The patient was transplanted two months later.

IRE is another modality that can be used in treating liver tumors, especially when close to adjacent major blood vessels or biliary ducts, as its not subjected to the heat sink effect. Results from clinical trials, and meta-analysis showed that IRE was effective and relatively safe (Table 2) [67,68,69,70,71,72]. Figure 9 discusses the management of a patient with cirrhosis and HCC with both MWA and IRE.

HIFU has been shown to be safe and effective with comparable outcomes to the other modalities [73,74,75,76,77,78]. A prospective comparative study revealed that HIFU was as safe and effective as RFA [79]. The new #HOPE4LIVER clinical trial is currently in the process of seeking regulatory approval to determine the efficacy of histotripsy for both primary and metastatic liver tumors.

Percutaneous ethanol or acetic acid injections are also sometimes used in resource-limited settings but are not usually recommended if other modalities are available. However, the literature is inconsistent, and some studies show a comparable role of ethanol in small liver tumors [31,80,81].

**Table 2:** Comparison of ablation modalities for hepatocellular carcinoma.

Authors	Study Design	Recruitment Years	Country	Sample Size	Comparison	Cancer	Residual Disease	LTR	OS	PFS	Complications	Mean Ablation Time
Kamal et al. [13]	RCT	2017	Egypt	56	MWA vs. RFA	≤5 cm HCC	5.9% vs. 11.2% (*p* = 0.673)	9.1% vs. 9.1% (1 year, *p* = 1.000)	82.1% vs. 78.6% (1-year, *p* = 1.0)	92.3% vs. 90.9% (*p* = 0.932)	7.1% vs. 0% (NSD)	4.41 vs. 14.21 min (*p* < 0.001)
Shibata et al. [58]	RCT	1999–2000	Japan	72	MWA vs. RFA	≤4 cm HCC	11% vs. 4% (*p* = 0.26)	17.4% vs. 8.3% (27 months, *p* = 0.20)	100% vs. 100% (27 months, *p* = 1.00)	N/A	11.1% vs. 2.8% (*p* = 0.36	33 vs. 52 min (*p* < 0.001)
Vietti et al. [55]	RCT	2011–2015	Switzerland	144	MWA vs. RFA	≤4 cm HCC	5% vs. 4% at 1 month (*p* = 0.93)	6% vs. 12% (48 months, *p* = 0.27)	86% vs. 84% (2-year *p* = 0.87)	NSD, *p* = 0.80	2 grade 4 in MWA vs. 3 grade 3 in RFA	4–6 vs. 12 min
Abdelaziz et al. [53]	RCT	2009–2014	Egypt	111	MWA vs. RFA	≤5 cm HCC	Complete ablations: 96.1% vs. 94.2% (*p* = 0.60)	3.9% vs. 13.5% (48. months, *p* = 0.49)	62% vs. 47.4% (2-year, *p* = 0.49)	N/A	3.2% vs. 11.1% (minor, *p* = 0.09)	N/A
Yu et al. [54]	RCT	2008–2015	China	203	MWA vs. RFA	≤5 cm HCC	Effectiveness: 99.6% vs. 98.8% (*p* = 0.95)	N/A	81.9% vs. 81.4% (3-year, *p* = 0.91)	N/A	3.4% vs. 2.5% (major, *p* = 0.59)	9 vs. 24.4 min (*p* < 0.001)
Chong et al. [56]	RCT	2011–2017	China	93	MWA vs. RFA	≤5 cm HCC	4.3% vs. 2.2% (1 month, *p* > 0.999)	N/A	72.7% vs. 67.1% (3-year, *p* = 0.899)	DFS: 24.1% vs. 22.7% (3-year, *p* = 0.912)	2.1% vs. 2.2% (overall, *p* > 0.999)	12 vs. 24 min (*p* < 0.001)
Wang et al. [66]	RCT	2008–2013	China	360	Cryoablation vs. RFA	≤4 cm HCC	Effectiveness: 98.5% vs. 95.8% (*p* = 0.106)	5.6% vs. 10% (*p* = 0.115)	67% vs. 66% (3-year, NSD)	DFS: 54% vs. 50% (3-year, NSD)	3.9% vs. 3.3% (major, *p* = 0.776)	N/A
Chen et al. [82]	Retrospective population-based	2004–2015	United States	3614	Cryoablation vs. RFA	HCC	N/A	N/A	NSD	NSD in CSS	N/A	N/A
Meijerink et al. [67]	RCT	2014–2018	Netherlands	51	IRE	CRLM ≤ 5 cm	74% achieved local tumor control after repeat procedures	32% after 1 year	Median 2.7 years (95% CI: 1.6, 3.8)	68% (95% CI: 59, 84)	40% adverse effects	Median procedure time 2.43 h w/o anesthesia
Frühling et al. [68]	Nonrandomized clinical trial	2011–2014	Sweden	30	IRE	HCC and liver metastasis	21.1% at 3 months and 34.2% at 6 months	28.6% after both 3 and 6 months	56.7%	N/A	3.3% major, 20% minor,	-
Glassberg et al. [83]	Meta-analysis	2009–2017	N/A	28 studies	MWA vs. RFA	HCC and liver metastasis	N/A	LTP: RR = 0.70; *p* = 0.02	NSD in 1-, 3-, and 5-year OS	NSD in 1-, 3-, and 5-year DFS	RR = 1.05; *p* = 0.75	N/A

Abbreviations: RCT, randomized control trial; LTR, local tumor recurrence; OS, overall survival; PFS, progression-free survival; DFS, disease-free survival; CSS, cancer-specific survival; NSD, no significant difference; MC = Monte Carlo; RR, relative risk, LTP, local tumor progression; w/o, without; N/A, not applicable.

While ablation is generally accepted as a safe alternative for unresectable liver tumors, various complications may occur, with hemorrhage being one of the most common complications in patients treated with thermal ablation [84]. A systematic review of 15,744 patients found that morality ranged from 0% to 0.88% with a pooled morality of 0.15% for RFA and 0.23% for MWA [85]. Major complication rates were 4.1% for RFA and 4.6% for MWA while minor complication rates were 5.9% for RFA and 5.7% for MWA [85]. Some of the common major complications noted were intraperitoneal hemorrhage, portal vein thrombosis, biloma, bile duct injury, and liver dysfunction [85]. With regard to cryoablation, there is a concern for an increased risk of bleeding due to the multiple cryoprobes without the ability to cauterize or coagulate vessels [84]. Furthermore, cryoablation runs the risk of cryoshock and parenchymal crack in the liver [84]. While IRE initially ran into the risk of cardiac toxicity and arrhythmias, the introduction of cardiac synchronization with IRE has made it a relatively safe choice for liver ablation [86]. Most of the complications of IRE are primarily due to electrode placement, and IRE appears to be safer than thermal ablation when adjacent to critical structures like the bile duct, as thermal ablation tends to lead to portal vein thrombosis, necrosis, and bilomas [86]. Moreover, most of the complications of IRE in liver ablation tend to be mild or transient, such as fever, local pain, abdominal distension, ascites, nausea, and vomiting [87]. In the case of HIFU, ablation of small liver tumors, even in patients with advanced cirrhosis, is relatively safe, but current challenges that are still being addressed are targeting tumors in difficult locations, such as the liver dome, structures near the rib cage, structures near large blood vessels or the heart, and structures adjacent to hollow viscera [88].

## 5. Renal Tumors

While the conventional treatment for renal cell carcinoma (RCC) has historically been radical nephrectomy, efforts to reduce the invasiveness, such as with nephron-sparing surgeries, have developed over the years [9,89,90,91]. This change to less invasive procedures to preserve the renal parenchyma has also led to use of image-guided thermal ablations for renal tumors with promising outcomes in unresectable tumors [36,92,93,94,95,96]. Current national comprehensive cancer network (NCCN) guidelines currently have ablative techniques as one of the primary treatment options for stage I-III kidney cancer [97].

**Figure 10:**
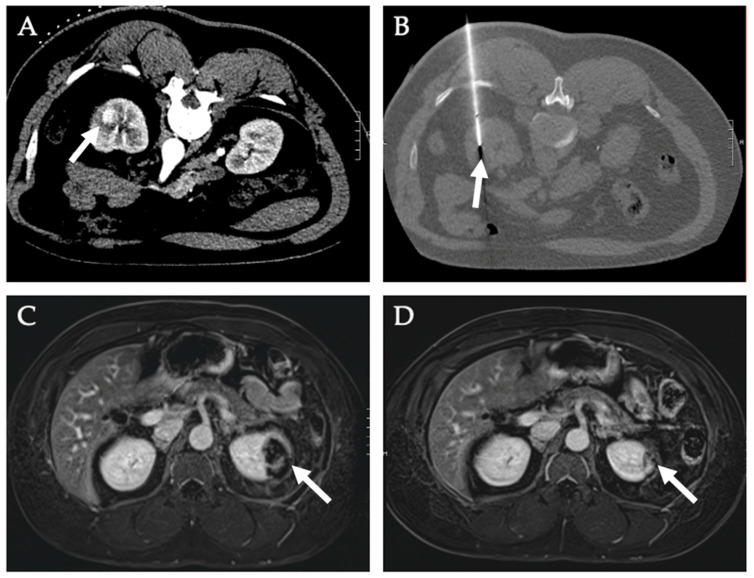
**Cryoablation of left renal mass.** A 57-year-old male with hematuria and left flank pain was found to have a left renal enhancing lesion (white arrow) on MRI images (**A**). (**B**) CT-guided percutaneous core needle biopsy found the 2.0 × 1.8 cm right renal lesion to be medullary fibroma while the 2.1 × 2.2 cm left renal lesion was International Society of Urologic Pathologists (ISUP)/World Health Organization (WHO) grade 2 RCC. The patient underwent cryoablation of the left mass. (**C**) One-month follow-up MRI showed complete tumor response. (**D**) Six-month follow-up MRI showed lesion shrinkage without residual tumor.

A meta-analyses and multiple cohort studies have shown that RFA and cryoablation have comparable efficacy and safety profiles for renal masses [98,99,100,101,102,103]. However, cryoablation appears to have better outcomes in larger >3–4 cm tumors than RFA [103,104,105,106]. Figure 10 presents the use of cryoablation on a patient with a left renal mass. MWA has also been shown to result in promising outcomes in renal tumors. A retrospective analysis of T1N0M0 RCC showed that the outcome of CT-guided percutaneous MWA is comparable to RFA or cryoablation with regard to treatment response and is associated with less sedation and lower treatment times [107]. Several other studies have shown comparable therapeutic and renal function outcomes among MWA, RFA, and cryoablation (Table 3) [108,109].

Complications for renal tumor ablation include both injury to the kidney, as well as the surrounding structures, like the vasculature or urinary tract. Complications include hemorrhage, ureteral stricture, urine leakage, urinary tract infections, pneumothorax, nerve injury, skin burns, and needle tract seeding [110]. In a prospective study of 573 renal ablation procedures (254 RFA and 311 cryoablation) performed in 533 patients with 633 tumors, complications did not statistically differ between the two [101]. In the RFA group, 3.9% had nerve injury, 2.1% had urothelial stricture, and 1.2% had hemorrhage/vascular injury/anemia [101]. In the cryoablation group, 4.8% had hemorrhage/vascular injury/anemia, 2.6% had hematuria, 1% had pulmonary embolus, and 0.6% had nerve injury [101]. A retrospective study of 105 US-guided percutaneous MWA in 111 patients with renal tumors found a complication rate of 24.8% with major complications including two hydrothorax and one bowel injury, while the minor complications included microscopic hematuria, mild thermal injury to the psoas muscle, perirenal hematoma, diarrhea, abdominal distension, edema of the lower extremities, and thermal injury to the pelvicalyceal system [111]. Some ways to prevent complications include holding anticoagulants to prevent bleeding, hydrodissection to avoid thermal injury to adjacent structure, and pyeloperfusion to protect the ureter [112,113].

**Table 3:** Comparison of ablation modalities for renal tumors.

Authors	Study Type	Inclusion Years	Sample Size	Comparison	Cancer	Findings
Thompson et al. [98]	Retrospective cohort	2000–2011	1803	Partial nephrectomy (PN) vs. RFA vs. cryoablation	T1N0M0 RCC	No significant difference in local recurrence-free survival. Metastases-free survival better in PN and cryoablation relative to RFA.
Atwell et al. [99]	Retrospective review	2000–2010	385	Cryoablation vs. RFA	RCC ≤ 3 cm	No significant difference in complications, local tumor recurrence, and local recurrence-free survival.
El Dib et al. [100]	Meta-analysis	2000–2008	883	Cryoablation vs. RFA	RCC	No significant difference in complications and pooled proportion of clinical efficacy
Atwell et al. [101]	Retrospective cohort	2000–2010	533	Cryoablation vs. RFA	RCC	No significant difference in major complication rates.
Andrews et al. [102]	Retrospective cohort	2000–2011	1798	PN vs. cryoablation vs. RFA	T1N0M0	No significant difference in survival and local recurrence, and metastases.
Zhou & Arellano [107]	Retrospective cohort	2006–2016	384	MWA vs. RFA vs. cryoablation	T1cN0M0 RCC	Similar complication rates and immediate renal function changes. MWA had lowest ablation time, procedural time, and dosage of sedative.
Martin & Athreya [108]	Meta-analysis	2003–2012	3153	Cryoablation vs. MWA	Small renal masses	No significant difference in primary effectiveness, cancer-specific survival, local tumor progression, and progression to metastatic disease.
Zhou et al. [109]	Retrospective cohort	2006–2016	297	MWA vs. RFA vs. cryoablation	T1aN0M0 RCC	At 2 years follow-up, no significant difference in local recurrence, metastatic progression, stability of renal function, and adverse event rate.

## 6. Pancreatic Tumors

Pancreatic cancer is the fourth leading cause of cancer-related death in the United States, and most patients are diagnosed with locally advanced or metastatic disease [114]. Current national guidelines recommend systemic therapy as the first-line treatment for unresectable pancreatic tumors [114]. However, most patients relapse, and chemotherapy is still associated with poor survival and complications [115]. Additionally, there are low rates of conversion surgery with R0 resections following systemic treatment [116]. Therefore, ablative techniques have emerged as an alternative adjuvant treatment for patients with pancreatic tumors, but they are mainly used as a consolidative treatment in stable tumors or as palliative care for tumors with persistent major vascular involvement [115,116]. The role of tumor ablation in pancreatic cancer is still understudied and there have been no completed trials that compare multiple ablation modalities [115,117]. Examples of the major ongoing trials are the “Pancreatic Locally Advanced Unresectable Cancer Ablation” (PELICAN) trial [118], which is an international multicentric superiority RCT investigating the effect of RFA in combination with chemotherapy in 228 patients across 16 centers in the Netherlands and four European centers, and the CROSSFIRE trial in the Netherlands, which compares the efficacy of IRE and chemotherapy (FOLFIRINOX) to the efficacy of FOLFIRINOX and stereotactic ablative radiotherapy (SABR) in 138 patients with non-resectable, non-metastasized, locally advanced pancreatic cancer (LAPC) [117].

**Figure 11:**
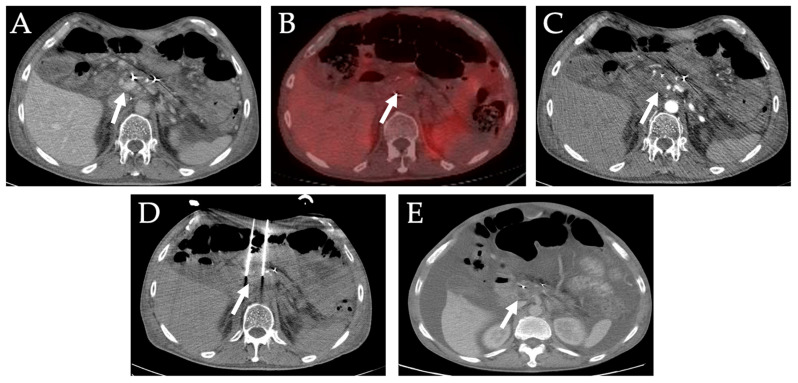
**Irreversible electroporation of locally advanced pancreatic mass**. A 57-year-old male with a history of pancreatic cancer presented with nonspecific epigastric pain. He was found to have locally advanced pancreatic head tumor (white arrow). He underwent chemotherapy and radiation and follow up CT and PET images (**A**–**C**) showed persistent locally advanced pancreatic head tumor. (**D**) He subsequently underwent IRE. (**E**). One-month follow up CT scan shows no residual disease.

The most used and studied ablative therapies for pancreatic cancer are RFA, IRE, and stereotactic body radiation therapy (SBRT) [117]. A recent systematic review screened 1037 articles published before 1 June 2014 and found 38 clinical studies with 1164 patients with LAPC, of which seven involved RFA, four involved IRE, 16 involved SBRT, five involved HIFU, two involved iodine-125, two involved iodine-125-cryosurgery, one involved photodynamic therapy, and one involved MWA [119]. The review found that all strategies seemed safe and feasible [119]. Of the modalities that had outcomes for postoperative-procedure-related morbidity and mortality, RFA had 4–22% and 0–11%, respectively, IRE had 9–15% and 0–4%, respectively, and SBRT had 0–25% and 0%, respectively [119]. Median survival for RFA, IRE, SBRT, and HIFU was 25.6, 20.2, 24.0, and 12.6 months, respectively [119]. Furthermore, the study found that IRE procedures were safer when done with a percutaneous approach than with an open approach [119]. Figure 11 describes the case of a patient with locally advanced pancreatic cancer who was treated with IRE.

Another review that examined ablation treatments for pancreatic cancer from January 2010 to May 2020, found 36 articles that met the inclusion criteria, of which 18 were for RFA, three for MWA, 11 for IRE, and four for electrochemotherapy (ECT) [115]. The mean (range) overall survival was 23 months (9–30), 24.9 months (4.9–85) and 11.5 months for RFA, IRE, and ECT, respectively [115]. The major and minor mean complication rate was 1.9% and 20.2% for RFA, respectively, 8.5% and 8.6% for MWA, respectively, 1.5% and 15% (open vs. 29% percutaneously) for IRE, respectively, and 0% and 23.1% for ECT, respectively [115].

The main risks of ablation in the pancreas are due to the location of the pancreas. Unlike other solid organs, the pancreas both involves and is surrounded by many medium- to large-sized blood vessels such as the celiac and hepatic arteries, the portal vein, and the superior mesenteric and splenic vessels [120]. Common complications due to ablation include pancreatitis, pancreatic duct fistulas and leaks, and pseudocysts. For RFA, there appears to be a correlation between the temperature reached in the ablation and complications, which is why some authors suggest avoiding over 90 °C in ablation temperatures [121]. Common complications associated with RFA also tend to include pancreatic fistulas, portal thromboses, and pancreatitis [121]. While the data concerning MWA complications is more sparce, possible complications include mild pancreatitis, hyperamylasemia, pancreatic ascites, mild hemorrhage, and pseudocysts [121,122]. Possible complications for cryoablation include pancreatic and bile leak, gastrointestinal bleeding and obstruction, infection, and hemorrhage [122].

## 7. Primary and Metastatic Adrenal Tumors

While ablation has been primarily focused on treating malignancies in organs such as the liver and kidneys, ablation modalities have expanded to target both benign and malignant endocrine tumors, especially those of the thyroid, parathyroid, and adrenal glands, to address systemic endocrinopathy [123]. Adrenal tumors have an estimated prevalence of 3–10% in those aged > 50 years and many are found as “incidentalomas” on abdominal imaging, for which most have no clinical significance [123,124,125]. Surgery remains the recommended treatment of choice for adrenal tumors that have clinical significance [123]. Ablation of these tumors is beginning to garner attention in both primary and metastatic settings due to its minimally invasive profile and its ability to be used in patients who are unfit for surgery. Furthermore, the expansion of ablation technology has allowed for further precision to target diseased tissue and preserve the adrenal parenchyma. This expansion can also be seen in new methods, such as the recent novel protocol of single-session CT-guided percutaneous MWA without pre-procedure adrenergic blockade that showed successful ablation, symptomatic improvement, and no residual tumor at 3-month follow-up in two patients with symptomatic Cushing syndrome who were not surgical candidates [126]. However, the data on clinical efficacy is still rather limited.

**Figure 12:**
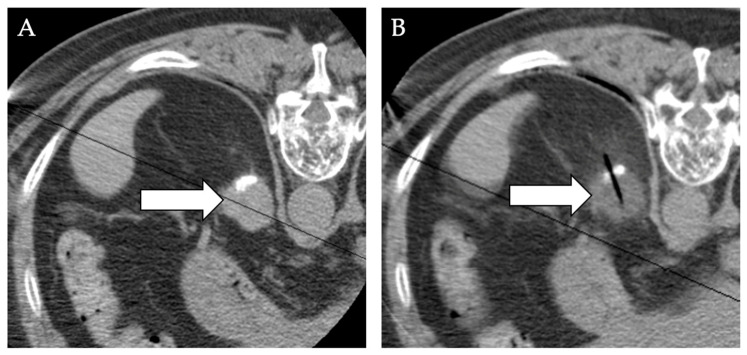
**Cryoablation of adrenal tumor**. (**A**) Axial CT image in an HCC patient with a single metastatic focus to the left adrenal (white arrow). (**B**) During cryoablation the entire mass (white arrow) demonstrates lowered HU as its density decreases during freezing. The “ice-ball” is difficult to visualize in fatty regions because its CT density is very close to that of fat. The peri-adrenal fat becomes “fuzzy” indicating extension of the “ice-ball” around the adrenal mass.

Most of the evidence of ablation therapy for adrenal tumors comes from small observational studies and case series that use RFA, MWA, and cryoablation. Figure 12 demonstrates a case of an adrenal tumor treated with cryoablation while still limited, the data is encouraging for ablation. In a recent review, Donlon and Dennedy found that unilateral aldosterone-producing adenomas (APA) and cortisol-secreting adenomas cured endocrinopathy with RFA and MWA for 75–100% of cases after a single ablation and 100% after repeated ablation [123]. They also found that thermal ablation leads to the promising resolution of hypertension following a thermal ablation for APA, similar to unilateral adrenalectomy [123]. Furthermore, this review also found promising results for RFA and MWA in metastatic adrenal tumors, in which the presence of residual tumor following ablation was seen in <25% of cases with <25% recurrence rates [123]. RFA has also shown to be promising in patients with severe adrenocorticotropic hormone (ACTH)-dependent Cushing syndrome for which bilateral adrenalectomy is not a suitable option [123,127]. A small case-series showed that all five patients who underwent bilateral RFA under CT-guidance had technical success with a resolution of their hypercortisolemia [127].

A recent 2021 meta-analysis studied the efficacy and safety of image-guided percutaneous ablation of adrenal metastases [128]. Of the 959 patients undergoing RFA, MWA, cryoablation, and ethanol injections, or some mixture of these modalities, they found a pooled 1-year local control rate of 80%, a pooled 1-year overall survival of 77%, an overall rate of severe adverse events (CTCAE grade 3 or higher) of 16.1%, and an overall rate of low-grade adverse events (CTCAE grade 2 or lower) of 32.6% [128].

Ablation also shows promise in metastatic pheochromocytomas and paragangliomas (PPGLI), which are neuroendocrine tumors [129]. A retrospective analysis performed at the Mayo Clinic analyzed 31 patients with metastatic PPGL and 123 lesions, 42 of which were treated with RFA, 23 with cryoablation, and four with PEI, from 1999 to 2017 [129]. Radiographic local control was obtained in 86% of lesions and 92% of the procedures led to improvement in metastasis-related pain or symptoms of catecholamine excess [129]. A total of 67% of the procedures had no complications, and 14%, 14%, 2%, and 2% of the procedures had Clavien–Dindo Grade I, II, IV, and V complications, respectively [129].

The complications and risks of adrenal ablation include hemorrhage, infection, and hypertensive crisis [130]. Thermal ablation modalities can lead to injury to surrounding organs like the kidneys, pancreas, and lungs. Hormonal activation, leading to stroke or cardiac syndromes from catecholamine release, and tumor seeding of the ablation probe tract are other risks of ablation [124,130].

## 8. Thyroid and Parathyroid Tumors

**Figure 13:**
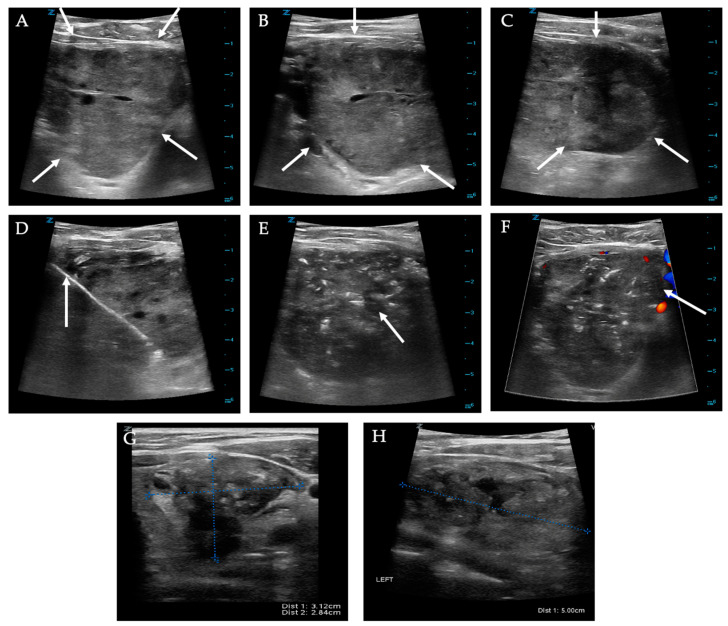
**Thyroid nodule radiofrequency ablation**. (**A**–**C**) Pre-ablation ultrasound images of solid nodule measuring 6.9 × 4.5 × 5.1 cm (white arrows). (**D**) Intraprocedure imaging shows trans-isthmic approach. (**E**,**F**) Post-ablation images show heterogeneous ablation changes throughout the nodule, and no internal vascularity. (**G**,**H**) One-month follow-up images show the nodule involuting and now measuring 5 × 3.1 × 2.8 cm.

While surgery is the main treatment option for patients with thyroid tumors, a growing number of studies have reported the safety and effectiveness of thermal ablations for thyroid tumors [131,132,133,134,135,136,137,138,139,140]. Several meta-analyses have also shown that thermal ablations are effective and safe alternatives [141,142,143,144] for primary and secondary hyperparathyroidism, primarily caused by parathyroid adenomas. Table 4 summarizes comparative studies on the various ablation modalities used, showing that RFA typically leads to superior volume reduction rates (VRR) for thyroid nodules when compared to other ablation modalities like MWA and laser ablation (LA). Comparative results of ablation modalities for parathyroid tumors are limited. Overall, ablation of thyroid and parathyroid tumors is becoming a more well-accepted alternative to surgery due to its minimal invasiveness and shorter recovery time [135,145]. Figure 13 discusses a representative case of a thyroid nodule ablation.

**Table 4:** Comparison of ablation modalities for thyroid and parathyroid tumors.

Authors	Study Type	Inclusion Years	Sample Size	Comparison	Cancer	Findings
Guo et al. [146]	Meta-analysis	2016–2019	1768 patients	RFA vs. MWA	Benign thyroid nodules	Similar pooled 3- and 6-month volume reduction rate (VRR), symptom improvement, cosmetic scores, and complications. RFA showed superior 12-month VRR.
He et al. [147]	Meta-analysis	1998–2015	873 patients	RFA vs. laser ablation (LA) vs. ethanol ablation (EA)	Benign thyroid nodules	RFA had the highest VRR. No significant difference in complication rate. RFA is most efficacious for solid or mostly solid nodules, EA for cyst or mostly cyst nodules.
Zheng et al. [148]	Meta-analysis	2012–2018	1461 patients	Cooled MWA vs. uncooled MWA	Benign thyroid nodules	Similar pooled 3-month VRR and pooled proportion of major complications. Uncooled MWA had higher overall and minor complications with more pain and skin burns.
Ha et al. [149]	Meta-analysis	2000–2013	184 patients	RFA vs. LA	Benign thyroid nodules	RFA was superior to laser ablation in reducing volume with fewer treatment sessions. No major complications with either.
Choi and Jung [150]	Meta-analysis	2014–2019	715 patients	RFA vs. LA vs. MWA	Primary papillary thyroid microcarcinoma (PTMC)	RFA had the highest mean VVR, followed by MWA and LA. Comparable safety profiles.
Suh et al. [151]	Meta-analysis	2008–2015	270 patients	RFA vs. EA	Locally recurrent thyroid cancer	RFA had a higher pooled VRR and pooled proportion of complete disappearance than EA. No significant difference in complication or recurrence rates.
Tong et al. [152]	Meta-analysis	2005–2017	1187 patients	RFA vs. MWA vs. LA	PTMC	No significant difference in VVR, proportion of complete disappearance and recurrence, and in major complications rate.
Cho et al. [153]	Meta-analysis	1999–2018	1208 patients	RFA vs. LA	Benign thyroid nodules	RFA had superior VRRs with less regrowth and delayed surgery. Comparable complication rates.
Trimboli et al. [135]	Meta-analysis	2002–2019	3195 nodules	RFA vs. LA	Benign non-functioning solid thyroid nodules	While both were effective in reducing volumes (maintained up to 2–3 years), RFA had superior VRRs.
Yang, Hsu, and Liou [154]	Meta-analysis	1994–2020	1514 patients	EA vs. RFA vs. polidocanol sclerotherapy vs. simple aspiration	Benign thyroid cystic nodules	No significant difference in VRR and therapeutic success rate between EA and RFA. EA had a higher pooled VRR than other modalities.
Wei et al. [155]	Multicenter retrospective cohort	2015–2020	119	RFA vs. MWA	Primary hyperparathyroidism	No significant difference in cure rates at 6 months and overall complication rates.

Abbreviations: LTR, local tumor recurrence; OS, overall survival; PFS, progression-free survival; NSD, no significant difference.

While ablation is generally safe for thyroid and parathyroid tumors, there are several risks and complications that may arise. The typical ones include hoarseness, hematomas, hypothyroidism, nerve injuries (especially to the laryngeal nerve), nodule rupture, skin burns, and hypocalcemia [156,157].

## 9. Prostate Tumors

The recent increase in screening measures has led to the detection of prostate cancers at earlier stages, calling into question the current standard treatments of radical prostatectomy, high dose prostate brachytherapy, and external beam radiation therapy that target the whole-gland prostate and reduce quality of life [158,159]. New treatments have been developed that aim to achieve both tumor control and functional preservation by partial ablation of the prostate while sparing the structures crucial for genitourinary function [158].

**Figure 14:**
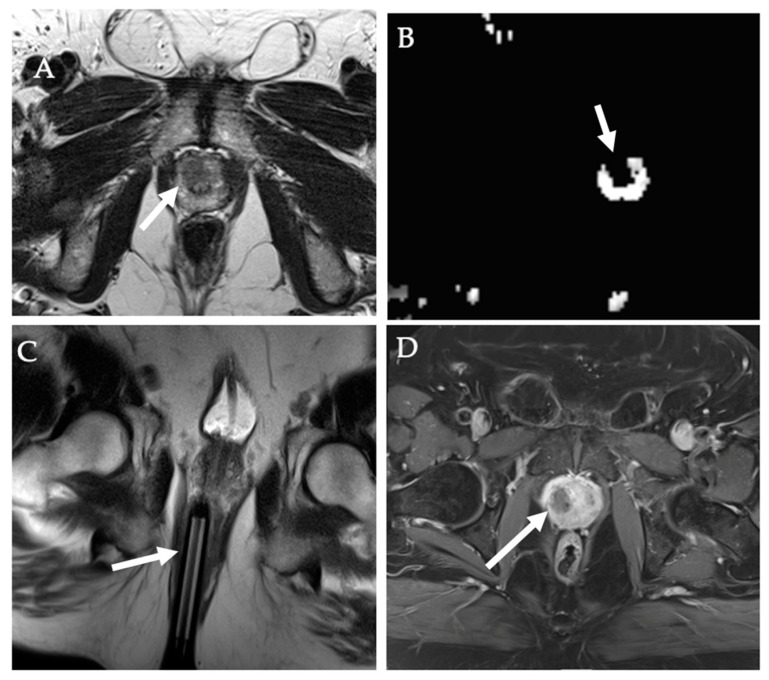
**Laser ablation of prostate tumor**. (**A**,**B**) Axial T2WI (**A**) and ADC (**B**) MR images show a right transition zone prostate lesion (white arrow) in a 66-year-old male with rising PSA and MR Bx positive for a Gleason score of 3 + 3 = 6 cancer. MR-compatible 18G biopsy gun deployed into the target during in-bore MR biopsy (**C**). (**D**) Subsequent MR-guided focal laser ablation was performed, with post-ablation contrast-enhanced T1WI showing complete necrosis of the target tumor.

The use of laser ablation for prostate tumors is gaining traction as it allows for the precise delivery to ablate tumors via coagulative necrosis while sparing prostate parenchyma. Results from a phase I clinical trial in eight men with intermediate-risk prostate cancer showed successful ablation with MR-guided focal laser ablation [160]. No grade 3 or higher adverse events, changes in International Prostate Symptom Score, nor International Index of Erectile Function 5 occurred at 6 months [160]. Seven men had their prostate specific antigen (PSA) decrease, and follow-up magnetic-resonance–ultrasound fusion biopsy did not detect residual disease in the ablation zone in five men but did find cancer outside the treatment margin in six men [160]. These results indicate the potential need to increase treatment margins. Promising results were seen in 120 patients with low-to intermediate-risk prostate cancer who were treated with focal laser ablation, showing no changes in sexual and urinary functional scores, a decrease in PSA, and low morbidity [161]. A positive biopsy for clinically significant prostate cancer post-ablation was seen in 18 (15%) patients [161]. Figure 14 discusses the case of a patient with a prostate tumor treated with laser ablation.

**Figure 15:**
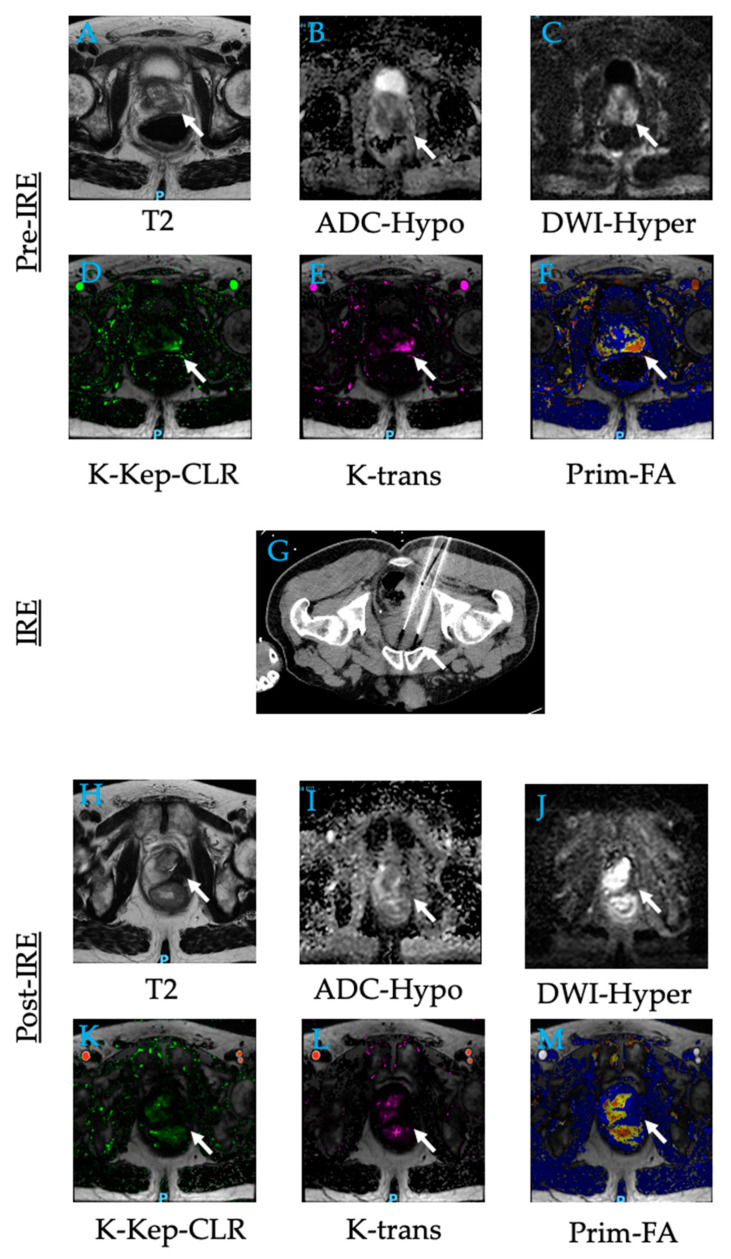
**Irreversible electroporation of left prostate adenocarcinoma.** A 64-year-old male with elevated PSA for which he underwent biopsy that showed adenocarcinoma in the left base of the prostate (white arrow) with Gleason score 7 and 8. (**A**–**F**) Contrast MR image of the prostate demonstrated a mass in the left peripheral zone. (**G**) He underwent IRE for the left side of his prostate. (**H**–**M**) Follow-up MR images show no residual tumor after 12 months. Abbreviations: ADC, apparent diffusion coefficient color-coded perfusion maps; DWI, diffusion-weighted imaging; DCE, color-coded dynamic contrast enhancement perfusion maps including K-Kep-CLR, K-trans, and Prim-FA.

HIFU is the most well-studied ablative modality used for prostate tumors and has shown to be effective for posterior lesions [158,162]. A prospective clinical trial reported that hemiablation HIFU therapy had promising functional and oncological outcomes in patients with localized, unilateral prostate cancer [163]. IRE is another focal therapy modality that has been gaining recent attention in men with prostate cancer. A recent meta-analysis demonstrated that IRE preserves urinary and erectile function at relatively high rates while also being safe with good oncologic outcomes. Figure 15 discusses the case of a patient with a prostate tumor treated with IRE. Other ablation techniques for prostate tumors include cryoablation, vascular-targeted photodynamic therapy (VP), RFA, MWA, and brachytherapy, but further comparative studies are needed.

While studies have shown the promise of ablation for prostate tumors, one of the present challenges in its adoption is adequate patient selection and precise disease localization [164,165]. The widespread adoption of multi-parametric MRI (mpMRI) has recently improved the detection of clinically significant prostate cancer, as evidenced by the PROMIS and PRECISION trials [166,167]. The mpMRI guidance has expanded the use of ablative modalities in prostate tumors, especially with transrectal ultrasound (TRUS)-MRI fusion for lesion targeting [165].

While generally safe, there are several risks of ablation of the prostate. Most complications typically occur within the first month of ablation and include hematuria, urinary tract infection, pain and discomfort, erectile dysfunction, dysuria, and urethral sloughing [168]. Recto-urethral fistula is a potential complication but a rare one and most often occurs when focal therapy is administered in a salvage setting and when the tumor is in the posterior portion of the prostate with extracapsular extension [168].

## 10. Primary and Metastatic Lung Tumors

**Figure 16:**
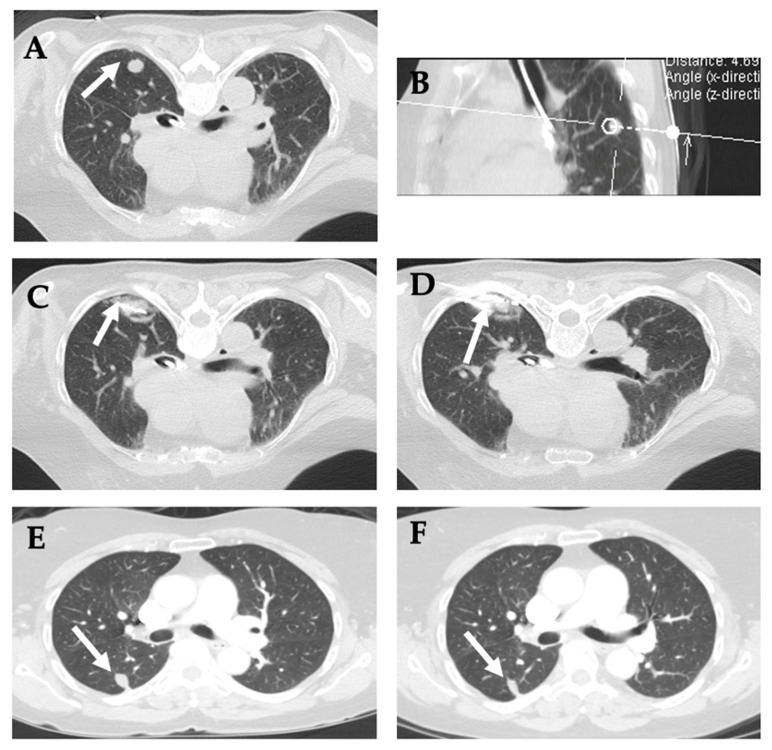
**Cryoablation of right lower lobe lung metastatic lesion suspected for colorectal cancer metastasis**. (**A**,**B**) CT images of the chest show the 1.2 cm nodule (white arrow) in the right lower lobe of the lung. (**C**) Under CT image-guidance, two Endocare PCS-17R cryoablation probes were advanced via a posterior approach, which bracketed the tumor to optimize the ablation coverage of the lesion. (**D**) Under CT image-guidance, a 19-gauge needle was advanced down to the lesion. An image was obtained and placed into the medical record. Samples were obtained for evaluation. Cryoablation was performed with three freeze cycles that lasted three, seven, and ten minutes, respectively, interposed with three minutes passive thaw cycles. (**E**) 6 and (**F**) 12-month follow up images show shrinking scar.

Lung cancer is the leading cause of cancer death for both men and women worldwide [169]. While radical resection is the treatment of choice for patients with lung cancer, only about 20–30% of patients are operable [170]. Image-guided thermal ablation is one of the many non-surgical treatments that patients with unresectable lung cancer can have. The most widely used ablative techniques for lung cancer are RFA, MWA, and cryoablation [171]. Table 5 summarizes most comparative studies that have been carried out presently, showing that RFA and MWA are comparable regarding outcomes and safety, while cryoablation still needs to be studied further. Figure 16 discusses the case of a patient with right lower lobe lung lesion treated with cryoablation. Very few studies address primary lung cancer alone and combine primary tumors with pulmonary metastases.

While ablation is generally safe in patients with lung cancer with a mortality rate <1%, it has the potential to lead to multiple complications, including pneumothorax, pleural effusion, and parenchymal hemorrhage [177]. Potentially fatal complications typically include major hemorrhage, pneumothorax that becomes intractable due to bronchopleural fistula, pulmonary artery pseudoaneurysm, formation of systemic air embolism, and pneumonitis [177]. A single center’s experience with 1000 RFAs in 420 patients found four deaths related to RFA, of which three patients died of interstitial pneumonia and one of hemothorax [178]. The major complication rate was 9.8%, and frequent complications include aseptic pleuritis (2.3%), pneumonia (1.8%), lung abscess (1.6%), bleeding that required blood transfusion (1.6%), pneumothorax that required pleural sclerosis (1.6%), bronchopleural fistula (0.4%), brachial nerve injury (0.3%), tumor seeding (0.1%), and diaphragm injury (0.1%) [178]. A single center’s experience with 204 MWAs in 184 patients found a major complication rate of 20.6%, in which 15.7% of cases resulted in pneumothorax, 2.9% of cases resulted in pleural effusions requiring chest tube placements, and 2.9% of cases resulted in pneumonia [179]. With regard to cryoablation, the most common complications include pneumothorax, hemoptysis, pleural effusion, injury to the phrenic nerve, and implantation of the tumor [180].

## 11. Primary and Metastatic Bone Tumors

Bone is a common site of metastasis and lesions here are often painful and can lead to many complications like fractures, hypercalcemia, and spinal cord compression [181,182,183]. While surgical resection and bone curettage are the primary treatment for bone tumors, not all tumors can be accessed surgically and the can often impact the quality of life by affecting ambulation and causing pain [184]. Recent attention has been placed on minimally invasive techniques that can improve both quality of life and local tumor control in patients with bone tumors [184,185]. These techniques include percutaneous thermal ablation like RFA, MWA, and cryoablation that are becoming well-established as safe and effective treatment options for primary and metastatic tumors [182,186].

A 2019 meta-analysis involving RFA, MWA, cryoablation, and magnetic-resonance-guided focused ultrasound (MRgFUS) found that all techniques resulted in pain relief after 1 and 3 months, in up to 91% and 95% of patients, respectively, though MRgFUS was found to have a noteworthy complication rate while the others were relatively safe [186]. While more comparative studies are needed, several systematic reviews, clinical trials, and cohort studies have shown the safety and effectiveness of RFA, MWA, and cryoablation for primary and metastatic bone tumors [184,187,188,189,190,191,192,193,194,195].

**Figure 17:**
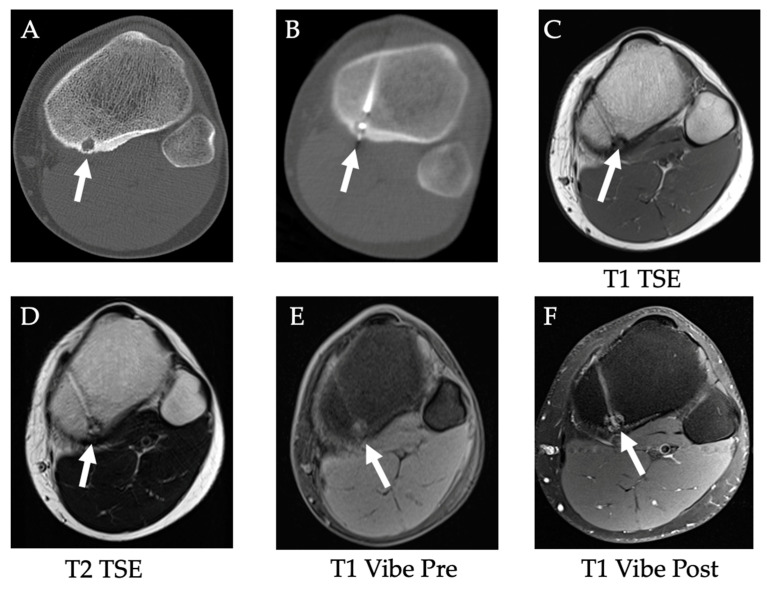
**Radiofrequency ablation of osteoid osteoma**. (**A**) A well-corticated osteoid osteoma with hypodense nidus (white arrow) in the posteromedial tibial metaphysis, (**B**) treated with RFA. (**C**–**F**) 5-month follow-up MR images show treated osteoid osteoma without residual lesion.

Each of the primary ablation modalities has specific clinical indications and established clinical applications. With regards to RFA, it can be used to treat benign tumors and has been advocated as a first-line treatment for spinal osteoid osteomas (OOs) and osteoblastomas (OBs) as it is associated with improved pain scores and quality of life [196]. RFA is also effective for managing painful primary and metastatic bone tumors, especially those ≤2 cm in size, as well as lytic tumors with neoangiogenesis and mechanical instability [196]. Figure 17 discusses the case of a patient with a well-corticated osteoid osteoma in the posteromedial tibial metaphysis treated with RFA.

Cryoablation is useful for bone tumors in that it can produce very large ablation zones with less pain in contrast to other modalities. It is also favored in sclerotic metastases compared to RFA due to the insulating effects of cortical bone. However, cryoablation is limited in that the ice ball size cannot be visualized in dense bone, and it can be associated with technical difficulty for tumors close to vital structures like the spinal cord or nerves. It is favored in sclerotic metastases when compared to RFA due to the insulating effects of cortical bone. Furthermore, cryoablation has fewer neural complications as it does not require electrical changes [196].

MWA has faster coagulation times, deeper penetration, and experiences reduced osseous impedance when compared to other ablative modalities, making it useful for deep and large lesions with less heat sink and charring buildup effects [196]. However, manufacturer guidelines are mainly calibrated for soft tissue tumors; hence, parameters for bone tumors need further optimization [197].

Interstitial laser ablation is also used in bone tumors and offers great precision with minimal effects to adjacent tissues and reduces costs for chemoprotection with extraspinal bone procedures [196]. This modality also preserves the overlying skin, providing a favorable cosmetic outcome.

MRg-HIFU is a noninvasive modality that has been used for OO and OB as well as bone metastasis, multiple myeloma, plasmacytoma, and other diseases [196]. It is particularly useful for flat bones with thin cortices (e.g., the iliac bone and scapula) with a low-volume disease load due to the insulating properties of cortical bone [196]. They are best suited for lesions between 1 cm and 12 cm as they cannot be too close for risk of skin burns and cannot be too deep given the poor penetrability of US [196].

Ablation for bone tumors is generally safe. In a retrospective study of 169 patients undergoing RFA for 217 tumors, the major complication rate was 2.3% (five patients) with four patients having secondary fractures [198]. The minor complication rate was 27.7% (60 patients) with the most frequent complication being immediate postoperative pain [198]. Other risks to ablating bone tumors include cortical loss and cement leakage [199]. This leakage is particularly more vulnerable when osteoplasty is done with cryoablation as the cooler temperature delays cement polymerization [199]. In contrast, thermal ablation modalities can accelerate cement polymerization and prevent further injection to strengthen the bone [199]. Other complications include inadvertent nerve and osteochondral injury near bone [199].

## 12. Primary and Metastatic Soft Tissue Tumors

Like bone tumors, soft tissue tumors can lead to chronic, severe pain and many complications, such as ulceration and mass effect [200]. While many soft tissue tumors are radiosensitive, a lot of them are not suited for radiation or surgery due to their location and adjacent structure, in which case, ablation remains an option. Percutaneous ablation can also prevent ulceration in certain soft tissue tumors of the skin or superficial locations [200].

**Figure 18:**
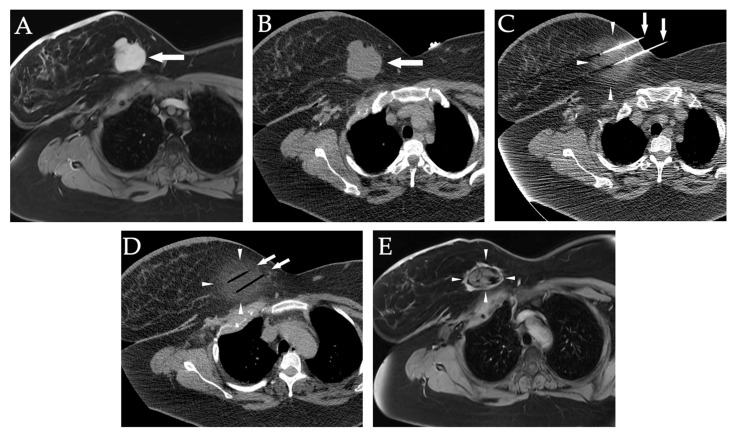
**Right breast sarcoma treated with CT-guided cryoablation.** (**A**) Axial gadolinium-enhanced T1-weighted, fat-suppressed MR image shows a lobulated right breast sarcoma mass (white arrow). (**B**) Axial pre-cryoablation, non-enhanced CT image shows target lobulated right breast lesion (white arrow). (**C**) Axial non-enhanced CT image during cryoablation shows two cryoprobes (white arrows) traversing the target lesion (white arrowheads). The lesion is less visible having become nearly iso-dense to fat due to freezing. (**D**) Axial non-enhanced CT image at the conclusion of cryoablation shows the two cryoprobe-ghost trajectories (white arrows) after the probes were removed (white arrowheads). (**E**) Axial gadolinium-enhanced T1-weighted, fat-suppressed MR image 2-years after cryoablation, shows a shrinking, non-enhancing, necrotic lesion surrounded by a thin rim of fibrous tissue (arrowheads).

The CRYODESMO-01 prospective, open-label non-randomized, non-comparative, multicenter trial found that cryoablation significantly improved functional status, reduced pain, and led to long-term disease control in non-abdominopelvic progressing desmoid tumors [201]. Cryoablation, as well as laser ablation, has also been shown to be effective as second-line treatments for certain symptomatic vascular anomalies [202]. Figure 18 demonstrates a case of a patient with right breast sarcoma treated with CT-guided cryoablation.

RFA has been shown to be effective for desmoid tumors, myeloma, soft tissue metastasis, and plasmacytomas [196,203]. Multiple studies have found RFA to be associated with high local tumor control rates with favorable overall survivals and minimal adverse events [204,205,206,207]. Currently, RFA is the most widely used technique for metastatic sarcoma [208].

Like in bone tumors, HIFU is also particularly useful in treating soft tissue tumors. It has been utilized for metastasis, multiple myeloma, plasmacytoma, and many other focal myeloproliferative disorders [196]. A multicenter retrospective analysis found that MRgFUS significantly reduced tumor volumes and pain in fifteen patients with extra-abdominal desmoid fribomatosis [209].

Complications of soft tissue tumors are similar to those of bone tumors, and most are due to the tumor’s location. If superficially located, these soft tissue tumors can lead to skin injury when ablated. Furthermore, there can be iatrogenic thermal damage to surrounding structures like nervous tissue, adjacent bowel, or other viscera [200].

## 13. Conclusions

Image-guided percutaneous ablation is becoming a well-established alternative to surgery in many different cancers as there is growing evidence of its effectiveness and safety profile in ablating both primary and metastatic tumors in various organs. Ablation allows for shorter recovery, less bleeding, and fewer risks than conventional treatment, and is transforming the treatment options available for patients. As this minimally invasive practice is becoming more widely adopted with frequent advancements in both technique and available ablation modalities, more efforts are needed to establish and update existing protocols to include ablation in the treatment of oncologic patients.

## Figures and Tables

**Table 1:** The advantages and disadvantages of the common ablation techniques.

Ablation Device	Advantages	Disadvantages
Ethanol	Cheap Fast and simple Well-tolerated	Non-uniform intertumoral distribution More recurrence Multiple treatments often required
RFA	Less expensive than other modalities Various electrode shapes Most-studied Widely available	Ablation zone not visible while ablating Pacemaker interference Painful (higher need for general anesthesia) Heat sink effect Requires grounding pads (Monopolar) Limited ablation zone
Cryoablation	Ablation zone visible while ablating No pacemaker interference Iceball visualization during CT, MR and US guidance	Inconsistent ablation sizes Some heat pump effect Long ablation times Higher risk of bleeding Cryoshock possibility
MWA	High temperatures (>150 °C) Large ablation zones Does not require grounding pads Less heat sink effect than RFA Short ablation times Can utilize multiple antenna probes in proximity	Usually performed under general anesthesia More difficult than RFA Not as efficient in larger tumors
Laser	Precise and efficient targeting Reduced image artifacts given lack of metal and small diameter of applicator	Limited energy penetration Small ablation zones (1–2 cm in diameter)
IRE	Short ablation times Well-defined ablation zones Adjacent tissue architecture preserved No heat sink effect	Requires general anesthesia with paralytic agents Risk of cardiac arrhythmias Challenging probe placement geometry
HIFU	Non-ionizing Non-invasive Extracorporeal	Requires patients to be still Near-field heating Long treatment times Can lead to skin side-effects
Histotripsy	Non-ionizing and non-thermal Can destroy tissue noninvasively Small scars No heat sink effect Tissue selectivity Well-demarcated boundaries Real-time feedback	Not widely available Low cavitation threshold in gas-containing organs makes it less suitable Not ideal for tumors within large tissue depth May induce thrombosis

**Table 5:** Comparison of ablation modalities for lung tumors.

Authors	Study Type	Inclusion Years	Sample Size	Comparison	Cancer	Findings
Chi et al. [172]	Retrospective Cohort + Meta-analysis	2003–2018	590	RFA vs. MWA	Primary and metastatic lung tumors	No significant difference in complication rates, complete ablation rates, median progression-free and overall survival
Macchi et al. [173]	RCT	N/A	52	RFA vs. MWA	Stage IV lung cancer	No significant difference in survival. MWA had significantly lower pain levels and a greater tumor size reduction
Bi et al. [174]	Meta-analysis	2004–2012	3095	RFA vs. SBRT	Stage I NSCLC	SBRT had significantly higher local tumor control rates. Comparable overall survival
Jiang et al. [175]	Meta-analysis	2004–2017	1840	RFA vs. MWA vs. cryoablation	Primary and metastatic tumors	RFA and MWA are more effective at controlling local progression rate than cryoablation. Comparable safety profiles across all three.
Yuan et al. [176]	Meta-analysis	2010–2017	3432	RFA vs. MWA	Primary and metastatic tumors	1–5 year overall survival higher in RFA. No significant difference in median local tumor progression free survival, complete ablation rates, and adverse events. RFA had higher median survival in metastatic tumors.

Abbreviations: LTR, local tumor recurrence; OS, overall survival; PFS, progression-free survival; NSD, no significant difference.

## Data Availability

Not applicable.
